# Characterization of phenolic compounds and evaluation of anti-diabetic potential in *Cannabis sativa* L. seeds: *In vivo, in vitro,* and *in silico* studies

**DOI:** 10.1515/biol-2022-1024

**Published:** 2024-12-31

**Authors:** Rafik El-Mernissi, Naoual El Menyiy, Amira Metouekel, Aziz Zouhri, Yahya El-Mernissi, Farhan Siddique, Sumaira Nadeem, Hassan Amhamdi, Oualid Abboussi, Abdulaziz Abdullah Alsahli, Mohammed Bourhia, Musaab Dauelbait, Gamal A. Shazly, Lhoussain Hajji

**Affiliations:** Bioactive and Environmental Health Laboratory, Faculty of Sciences, Moulay Ismail University, Meknes, B.P. 11201, Morocco; Physiology and Physiopathology Team, Faculty of Sciences, Genomic of Human Pathologies Research Centre, Mohammed V University, Rabat, Morocco; Laboratory of Pharmacology, National Agency for Medicinal and Aromatic Plants, Taounate, 34025, Morocco; University of Technology of Compiègne, EA 4297 TIMR, 60205 Compiègne Cedex, France; Applied Chemistry Research Unit, Faculty of Science and Techniques, Abdelmalek Essaadi University, Al-Hoceima, Tetouan, Morocco; School of Pharmaceutical Science and Technology, Tianjin University, Tianjin, P.R. China; Department of Pharmacy, The Women University, Multan, 60800, Pakistan; Department of Botany and Microbiology, College of Science, King Saud University, Riyadh 11451, Saudi Arabia; Laboratory of Biotechnology and Natural Resources Valorization, Faculty of Sciences, Ibn Zohr University, 80060, Agadir, Morocco; University of Bahr el Ghazal, Freedom Street, Wau, 91113, South Sudan; Department of Pharmaceutics, College of Pharmacy, King Saud University, Riyadh 11451, Saudi Arabia

**Keywords:** medicinal plant, natural products, bioactive compounds, nutraceuticals, biological properties

## Abstract

Moroccan *Cannabis sativa* L. seeds were investigated for their phenolic profile and antidiabetic potential. Ultra-high-performance liquid chromatography with diode array detection and electrospray ionization mass spectrometry analysis revealed a rich phenolic composition, including benzoic acid, cannabisin B, genistein, and epicatechin. *In vitro*, the seed extract exhibited potent α-amylase inhibitory activity (half-maximal inhibitory concentration = 25.02 ± 4.03 μg/mL). *In vivo* studies in diabetic rats demonstrated significant hypoglycemic, hypolipidemic, hepatoprotective, and nephroprotective effects. Molecular docking studies further supported these findings, revealing strong interactions between identified phenolic and the α-amylase enzyme. These results highlight the potential of *C. sativa* seeds as a natural source of bioactive compounds for diabetes management.

## 1 Introduction

In recent years, there has been a growing interest in exploring natural compounds, particularly polyphenols, for their potential therapeutic effects in managing diabetes mellitus a widespread chronic metabolic disorder characterized by persistent high blood sugar levels due to inadequate insulin synthesis or poor insulin utilization. This condition affects millions globally and poses a significant social and economic burden, with an estimated 1.5 million deaths attributed to diabetes each year [1,2]. The increasing prevalence of diabetes, along with its associated complications, places considerable pressure on healthcare systems. Chronic hyperglycemia can lead to microvascular complications such as diabetic retinopathy, nephropathy, and neuropathy [3,4]. Additionally, individuals with diabetes are at a significantly higher risk for macrovascular complications, including cardiovascular disease, stroke, and peripheral artery disease [5].

Current diabetes management strategies emphasize achieving glycemic control through a combination of pharmacological therapies, lifestyle modifications, and nutritional interventions. A variety of conventional hypoglycemic agents are available that function by supplementing insulin, improving insulin sensitivity, stimulating insulin secretion, and facilitating glucose uptake. However, these medications typically target a single mechanism of action and are often associated with undesirable side effects such as diarrhea, lactic acidosis, hepatic failure, weight gain, tachycardia, and hypothyroidism. Furthermore, the development of tolerance over time necessitates frequent dosage adjustments. Unfortunately, these medications do not provide a permanent cure for diabetes; they primarily focus on managing its symptoms [6,7].

In light of these challenges, there is an urgent need to explore innovative strategies that can address these limitations and offer more effective long-term management solutions for diabetes. In this context, *Cannabis sativa* L., commonly referred to as marijuana or hemp, has gained attention for its potential medicinal properties [8]. Historically used for various therapeutic purposes – including pain management and antioxidant effects – the therapeutic potential of cannabis in managing diabetes has gained traction in recent years [8,9,10,11].

In Morocco, *C. sativa* has long been illegal but tolerated to varying degrees. A significant shift occurred in June 2021 when the Moroccan parliament passed a landmark law regulating cannabis for medical, pharmaceutical, and industrial purposes. This legislative change not only signifies a progressive step toward addressing the societal and economic dimensions of *C. sativa* but also opens new avenues for scientific exploration and utilization of *C. sativa* L. in various therapeutic applications, including diabetes management [12]. Given this evolving landscape, investigating the antidiabetic properties of polyphenols derived from cannabis seeds emerges as a compelling avenue for research. Polyphenols are known for their antioxidant and anti-inflammatory properties and have shown promise in modulating glucose metabolism and improving insulin sensitivity – making them attractive candidates for diabetes management [13]. The unique composition of polyphenols in cannabis seeds such as cannabisins and cannaflavins [14,15] coupled with recent legal developments in Morocco and the country’s specific environmental factors presents an opportune moment to delve into the therapeutic potential of this natural resource.

In light of these considerations, this study aims to explore the phenolic profile and antidiabetic properties of Moroccan *C. sativa* L. seeds through a multifaceted approach encompassing *in vivo*, *in vitro*, and *in silico* methodologies. By elucidating the biochemical composition and pharmacological effects of cannabis seed-derived polyphenols, this research seeks to contribute valuable insights toward developing novel therapeutic strategies for diabetes management.

## 2 Materials and methods

### 2.1 Plant collection

Plant samples were collected in September 2021 from the Tafrant region, Taounate, Morocco (34°39′28.4″N, 5°05′58.9″W). Subsequent to the collection, the plants were air-dried in a shaded environment and the seeds were isolated. These seeds were then stored in airtight plastic bags under ambient temperature conditions (24–27°C) until further analysis.

### 2.2 Plant identification

A botanist at the Scientific Institute of Rabat identified the plant, and a voucher specimen labeled with the number RAB 112735 was archived in the institute’s herbarium.

### 2.3 Quality control of plant material of *C. sativa* L. seeds

#### 2.3.1 Oil content

*C. sativa* L. seeds after being cleaned and dried to achieve a consistent dry weight were processed by grinding using a mill. The oil content of the ground samples was assessed using a Soxhlet apparatus with hexane as solvent. The solvent was then evaporated using a rotary vacuum evaporator, and the oil content of the *C. sativa* L. seeds was quantified following a method outlined in the AOAC [16].

#### 2.3.2 Ash content

The ash content was assessed following the procedure outlined by Laaroussi et al. [17]. Initially, the *C. sativa* L. seeds underwent preliminary ashing at 500°C for 4 h, followed by measurement of the resulting ash content. The residue was subsequently weighed three times, and the percentage of ash (%) was calculated according to the formula:

\[{\mathrm{Ash}}( \% )=\frac{{\mathrm{masse\; of\; ash}}}{{\mathrm{masse\; of\; ground}}C.{sativa}{\mathrm{L}}.{\mathrm{seeds}}}.]\]

#### 2.3.3 Organic material content

The quantity of organic material was computed using the equation [17]:

\[{\mathrm{Organic\; material}}(\left \% )=100\left-{\mathrm{Ash}}(\left \% ).]\]

#### 2.3.4 Humidity level

The moisture content of *C. sativa* L. seeds was determined through a meticulous gravimetric analysis. This method entails precisely measuring the weight of the sample before and after drying in a convection oven at 105°C until a constant weight is achieved [16].

#### 2.3.5 pH determination

A mass of 5 g of the sample was mixed with 500 mL of distilled water. The mixture was stirred for 5 min at room temperature. The mixture was filtered, and the pH was measured using a pH meter [18].

### 2.4 Extract preparation

The study commenced with the air-drying and mechanical pulverization of fresh *C. sativa* L. seeds to produce a powdered form. A portion of 200 g of this powder was subjected to maceration with a 70% ethanol solution while being continuously stirred for 48 h at room temperature. Post-maceration, the resulting mixture underwent filtration utilizing Whatman filter paper No. 1 to remove any insoluble materials. The filtrate obtained was then concentrated at 40°C under vacuum conditions using the Buchi rotary evaporator R-250 to eliminate the solvent. The concentrated extract was subsequently subjected to freeze-drying to obtain the lyophilized extract, which exhibited a yield of 23% w/w. Following extraction, the obtained extract was carefully stored in a refrigerator (4°C) until it was required for further analysis and experimentation.

### 2.5 Experimental animal

Male albino Wistar rats, weighing between 190 and 250 g, were procured for this research from the animal facility of the Bioactives and Environmental Health Laboratory at the Faculty of Sciences, Moulay Ismail University, Meknes, Morocco. The animals were housed in Plexiglas cages under standard laboratory conditions, maintaining a temperature of 25 ± 2°C, humidity levels of 55 ± 5%, and a 12-h light/dark cycle. Throughout the experimental period, the rats had *ad libitum* access to both food and water. All animal care and experimental protocols adhered strictly to ethical guidelines as outlined in authorization number 86/609/EC20.


**Ethical approval:** The research related to animal use has been complied with all the relevant national regulations and institutional policies for the care and use of animals, and has been approved by the ethical committee of the Faculty of Sciences, Moulay Ismail University, Morocco.

### 2.6 Phytochemical screening

An assessment was conducted to screen for steroids/terpenoids, alkaloids, flavonoids, saponins, phenols, tannins, coumarins, and free quinone. This evaluation relied on observing color intensity or the formation of precipitates, which are recognized as key analytical indicators for these tests [19].

### 2.7 Identification of phenolic compound

Phytochemical analyses of cannabis seed extract were carried out using ultra-high-performance liquid chromatography with diode array detection and electrospray ionization mass spectrometry (UHPLC-DAD–ESI/MS), as described previously by Zefzoufi et al. [20]. The experimentation was conducted using an Ultimate 3000 system (Dionex, A, USA), which featured a quaternary pump (HPG 3400 RS), an autosampler (WPS 3000 TSL), and a column oven (TCC 3000). For the method, a Kinetex C18 reversed-phase column (250 mm × 4.6 mm, 2.6 μm particles) supplied by Thermo Fisher Scientific (CA, USA) was employed. The chromatographic separation was achieved using a gradient of solvent A (0.1% formic acid aqueous solution) and solvent B (methanol), with the following profile: 0–3 min, a linear gradient from 5 to 25% B; 3–6 min, held at 25% B; 6–9 min, a gradient from 25 to 37% B; 9–13 min, held at 37% B; 13–18 min, a gradient from 37 to 54% B; 18–22 min, held at 54% B; 22–26 min, a gradient from 54 to 95% B; 26–29 min, held at 95% B; 29–29.15 min, returned to initial conditions at 5% B; and from 29.15 to 36 min, held at 5% B. The flow rate of the mobile phase was maintained at 1 mL/min. Ultra-violet visible spectral data for all peaks were recorded in the range of 200–400 nm, while chromatographic profiles were monitored at 280 nm. The mass spectrometer utilized was a TSQ Endura (Thermo Fisher Scientific, CA, USA) triple quadrupole equipped with heated-electrospray ionization (H-ESI) source operating in negative mode. Nitrogen (purity >99.98%) served as the sheath gas, ion sweep gas, and auxiliary gas at flow rates of 65, 0, and 40 arbitrary units (a.u.), respectively. H-ESI vaporizer and ion transfer tube temperatures were set at 350°C. The electrospray voltage was maintained at 2.5 kV. For characterization and evaluation, the full scan MS acquisition mode (*m*/*z* 50–1,000) in Q1 with a mass resolution of 0.7 *m*/*z* full-width half maximum (FWHM) and a scan time of 0.5 s was primarily utilized.

### 2.8 Effect of hydro-ethanolic extract on alloxan-induced diabetic rats

#### 2.8.1 Diabetes induction and study design

Diabetes was triggered by administering a single intraperitoneal injection of 150 mg/kg body weight of alloxan monohydrate solution to albino Wistar rats that had been fasted for 12–14 h [21]. To avoid hypoglycemic shock following diabetes induction, the rats were provided access to 10% glucose solution bottles for the next 24 h. One week post-injection, blood glucose levels were measured, and animals were classified as diabetic if their fasting blood glucose level reached or surpassed 200 mg/dL.

Twenty-four selected animals, including both diabetic and non-diabetic subjects, were divided into four groups, each consisting of six individuals (*n* = 6):

- Group 1 normal healthy control consisted of six non-diabetic rats, who were administered distilled water orally at a dosage of 10 mL/kg of body weight per day.
- Group 2 diabetic control (DC) comprised six diabetic rats, who were orally administered a dosage of distilled water at 10 mL/kg of body weight per day.
- Group 3, treated group (TG) six diabetic rats were orally treated with a hydro-ethanolic extract of *C. sativa* L. seeds (CSSE) dissolved in distilled water at a dosage of 300 mg/kg of body weight per day.
- Group 4 (DG) involved six diabetic rats treated with glibenclamide, a reference drug, at a dosage of 2 mg/kg of body weight per day.

The study lasted for a total of 28 days, during which all treatments were administered orally once every morning via a feeding tube prior to the animals’ meals. Throughout the treatment period, body weight and fasting blood glucose levels were monitored by collecting tail blood samples and analyzing them with a glucometer (Accu-Chek Active-300) on days 0, 7, 14, 21, and 28. Changes in body weight were also recorded.

At the conclusion of the 28-day experimental period, the animals were anesthetized using diethyl ether and subsequently euthanized by decapitation. Blood samples were promptly collected for analysis of serum biochemical parameters, including AST, ALT, urea, creatinine, LDL, HDL, cholesterol (CT), and triglycerides (Trg), using an automated chemistry analyzer (KONELAB 20i: AUTO-ANALYZER).

#### 2.8.2 Oral glucose tolerance test (OGTT)

**Figure 1:**
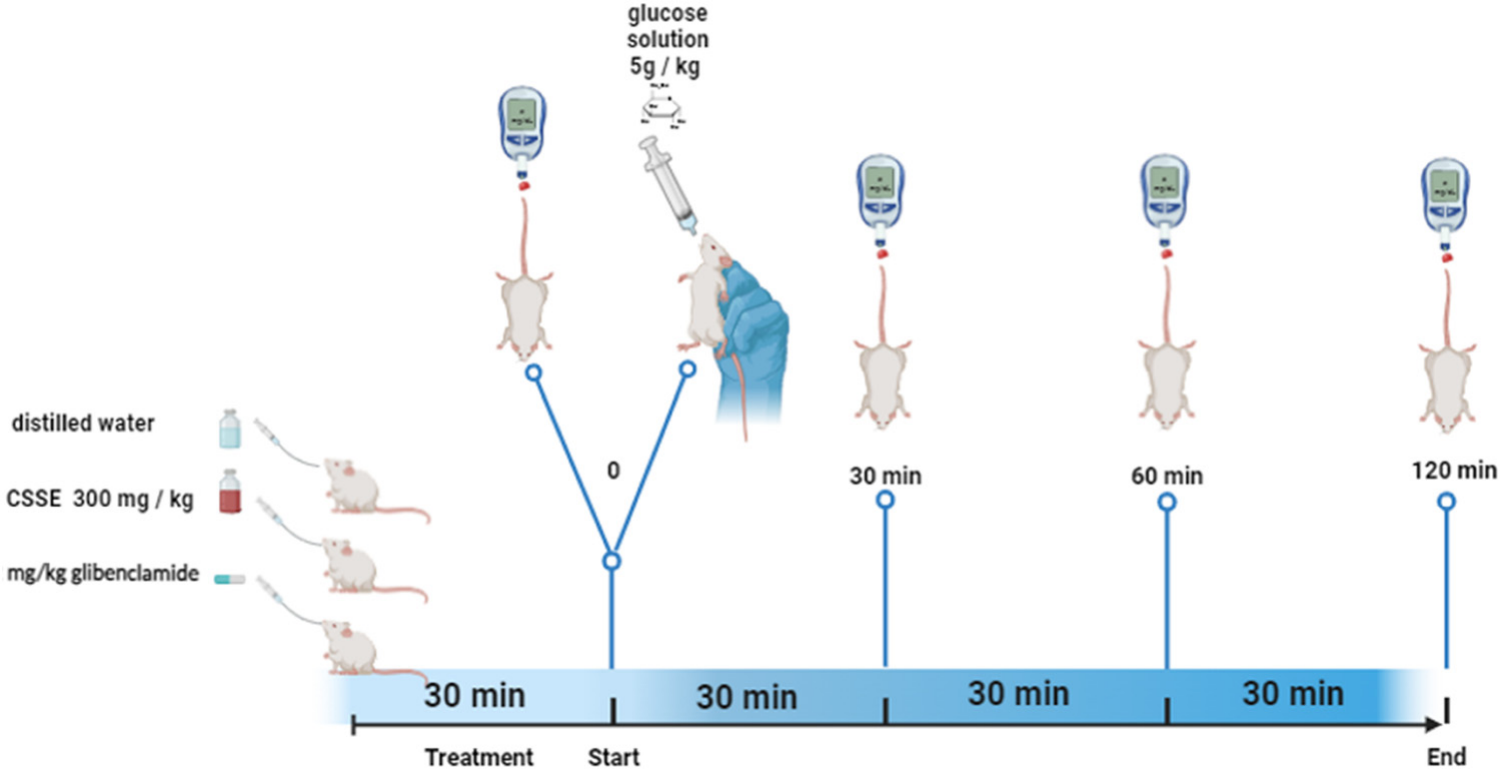
OGTT model.

OGTT was conducted following the procedure outlined by Patel et al. [22], with slight modifications as illustrated in Figure 1. Briefly, normal rats that had fasted for 12 h were divided into three groups, each consisting of six individuals (*n* = 6). Group 1 served as the control and received distilled water (1 mL/100 g body weight), Group 2 was treated with 300 mg/kg of hydro-ethanolic extract from *C. sativa* L. seeds (CSSE), and Group 3 received 2 mg/kg of glibenclamide. Thirteen minutes after treatment administration, the animals were given a glucose solution at a dosage of 5 g/kg. Blood samples were collected from the tail prior to dosing and at regular intervals of 0, 30, 60, 90, and 120 min following glucose administration.

#### 2.8.3 Inhibition of α-amylase activity *in vitro*

The α-amylase inhibition assay was conducted in accordance with the method outlined by Aazza et al., utilizing the starch-iodine test [23]. Briefly, the assay mixture consisted of 40 µL of 0.02 M sodium phosphate buffer (pH 6.9, containing 6 mM sodium chloride), 20 µL of α-amylase solution (50 µg/mL), and 20 µL of the extract or acarbose at varying concentrations. This mixture was incubated at 37°C for 10 min. Following this, 20 µL of soluble starch (0.5%, w/v) was introduced to each well, and the incubation continued at 37°C for an additional 15 min. To halt the enzymatic reaction, 20 µL of 1 M HCl was added, followed by the addition of 100 µL of iodine reagent (5 mM KIO₃ and 5 mM KI). Color changes were observed, and absorbance was measured at 620 nm using a microplate reader. The control sample, representing 100% enzyme activity, was prepared without the addition of the extract. The inhibitory activity (%) was determined using the following formula:

\[ \% {\mathrm{inhibition}}=1-\frac{({\mathrm{Abs}}2-{\mathrm{Abs}}1)}{({\mathrm{Abs}}4-{\mathrm{Abs}}3)}\times 100,]\]

with

- Abs1 is the absorbance of the incubated mixture that includes the extract, starch, and α-amylase.
- Abs2 refers to the absorbance of the incubated mixture with the extract and starch only.
- Abs3 represents the absorbance of the mixture containing starch and α-amylase.
- Abs4 is the absorbance of the incubated mixture with starch alone.

The half-maximal inhibitory concentration (IC_50_) was determined by curve-fitting using graphpadprism10 (10th version).

#### 2.8.4 Inhibition of α-amylase activity *in vivo*

To assess the *in vivo* α-amylase inhibitory activity of the *C. sativa* seed extract (CSSE), an oral starch tolerance test was employed [24]. Briefly, three groups of fasted rats, each consisting of five animals, were established. The control group received only distilled water orally (10 mL/kg), while the CSSE group was administered the extract at a dose of 300 mg/kg. The acarbose group received 10 mg/kg of acarbose. Thirty minutes post-treatment, all rats were orally loaded with starch (2 g/kg). Blood samples were collected from the tail vein of the rats at 0, 30, 60, and 120 min to measure blood glucose levels.

#### 2.8.5 Statistical analyses

It is expressed as mean ± SD, Statistical comparisons among the groups were conducted using a two-way analysis of variance, followed by the Tukey test, utilizing GraphPad Prism (10th version). Significance was considered at *p* < 0.05.

### 2.9 Glide molecular docking methodology

Analysis of the aqueous extract of hydro-ethanolic extract of *C. sativa* L. seeds showed the presence of seven polyphenols such as which served as the ligands to be docked via the maestro glide docking method with an α-amylase inhibitory (Protein Data Bank [PDB] ID: 4GQR) target proteins along with its respective co-crystallized ligand and the reference compound, acarbose.

#### 2.9.1 Protein preparation

The PDB repository (https://www.rcsb.org) [25] was the source of an α-amylase inhibitory (PDB ID: 4GQR) target protein. The bond orders were assigned followed by the addition of hydrogen atoms and finally disulfide bonds were created in the Glide (Maestro 12.8) [26] of the protein preparation wizard panel followed by optimization of protein structure with OPLS4. The receptor grid file was then set to create the ligand binding pocket.

#### 2.9.2 Ligand preparation

All the ligands were sketched by ChemDraw 20.1.1 followed by energy minimization via Chem3D 20.1.1 [27]. LigPrep module was incorporated to fetch sdf file format and finally prepared ligands for molecular docking (Maestro 12.8). The correct chiralities were used to create low-energy 3D structures such that at a physiological pH of 7.2 ± 0.2 and the potential ionization states were created for all of the retrieved ligand structures.

#### 2.9.3 Receptor grid generation (RGG)

For ligand docking, RGG assists in the size and position parameters of the probable active site of the target protein. With the use of the RGG tool of Schrodinger Maestro 12.8, the scoring grid was expressed based on the binding of co-crystallized ligand in the target protein. Non-polar receptor atoms’ van der Waals (vdW) radius scaling factor was affixed to 1.0 whereas the partial charge cut-off was set at 0.25.

#### 2.9.4 Protein–ligand docking

The created RGG file was employed to conduct molecular docking studies by using the Glide tool of Schrodinger Maestro 12.8 while standard precision was the factor to dock the already prepared ligands. For ligand atoms, the vdW radius scaling factor was scaled at 0.80 with a partial charge cut-off of 0.15.

## 3 Results

Significant causal relationships are evident among the results presented.

### 3.1 Quality control of plant material of *C. sativa* L. seeds

Table 1 outlines the quality control evaluations performed on *C. sativa* L. seeds, which revealed a moisture content of approximately 5%, an oil content of 28%, an ash content of 5.99%, a pH level of 5.3, and an organic material content of roughly 94.01%.

**Table 1:** Oil content, ash content, organic material content, humidity level, and pH of *C. sativa* L. seeds

*C. sativa* L. seeds	Oil content	Ash content	Organic material content	Humidity level	pH
	28 ± 1.9%	5.99%	94.01	5%	5.3

These findings emphasize the critical role of maintaining optimal seed quality for preservation and therapeutic applications. The low moisture content is especially advantageous as it reduces the risk of microbial growth and spoilage, thereby extending the shelf life of the seeds. Moreover, the substantial oil content indicates that these seeds could serve as a significant source of essential fatty acids, enhancing their nutritional and medicinal value.

The slightly acidic pH level of 5.3 helps to maintain the stability of bioactive compounds within the seeds, which is essential for preserving their efficacy. Additionally, the high organic material content highlights the seeds’ potential utility in various applications, such as nutritional supplementation and the development of functional foods.

Overall, these quality control assessments provide essential insights into the characteristics of *C. sativa* L. seeds, underscoring their value as a versatile resource for both agricultural and medicinal purposes.

### 3.2 Phytochemical screening

The initial phytochemical analysis of *C. sativa* L. seeds, conducted using a hydro-ethanolic extract, revealed the presence of various secondary metabolites, including steroids, terpenoids, flavonoids, saponins, phenols, tannins, coumarins, and quinones, while alkaloids were notably absent (Table 2). These findings suggest that *C. sativa* L. seeds serve as a rich reservoir of secondary metabolites. The significant presence of these phytochemicals underscores the importance of *C. sativa* L. as a medicinal plant.

**Table 2:** The qualitative analysis of *C. sativa* L. seeds hydro-ethanolic extract

Alkaloids	−−
Tannins	++
Phenols	++
flavonoids	++
Saponin	++
Coumarins	++
Carbohydrates	++
Terpenes	++
Quinones	++
Steroids	++

−− Absent, ++ present.

### 3.3 Identification of phenolic compound

**Figure 2:**
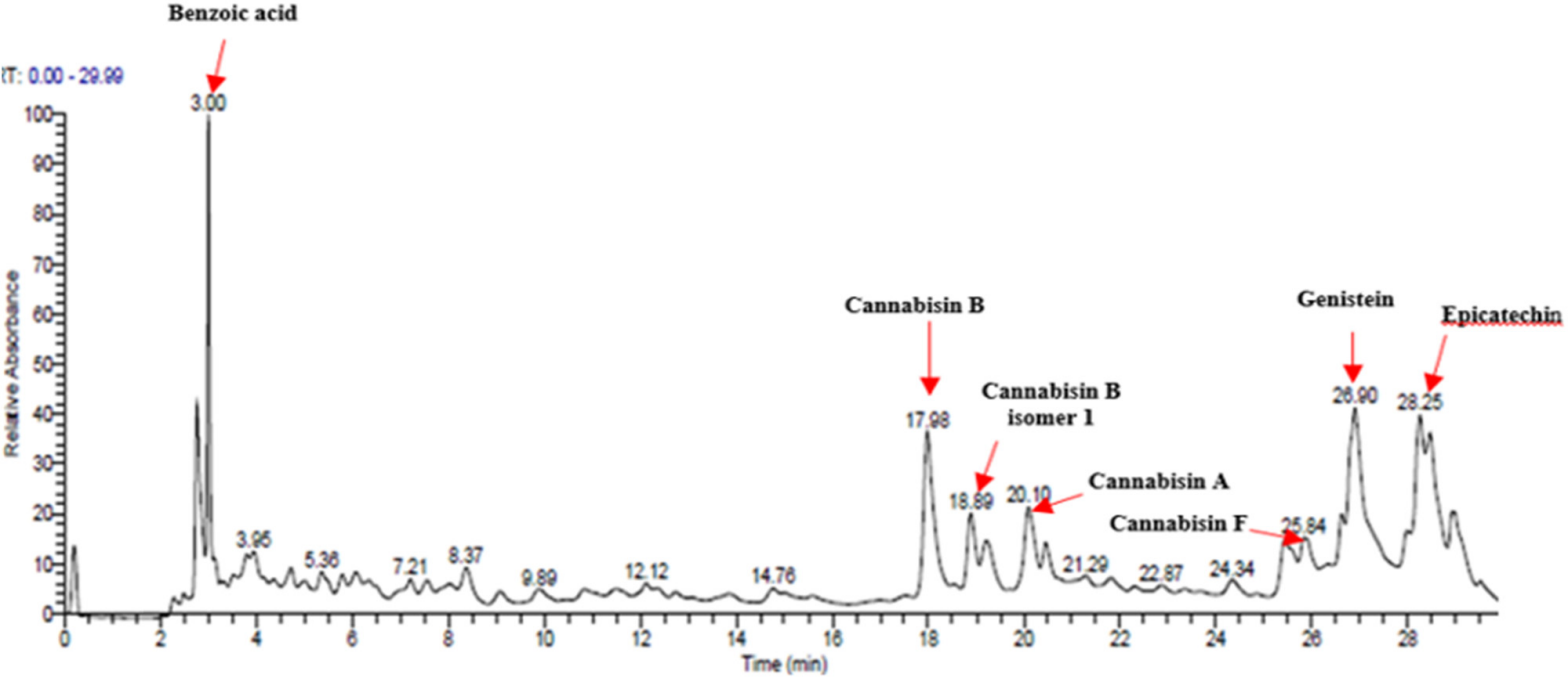
Chromatographic profile of CSSE obtained by UHPLC–ESI/MS at 280 nm.

The hydro-ethanolic extract from *C. sativa* L. seeds (CSSE) was subjected to qualitative analysis using UHPLC-DAD–ESI/MS) to identify the major components potentially involved in its anti-diabetic effects (Figure 2).

As detailed in Table 3 a total of seven phenolic compounds were successfully identified and categorized into two subgroups: phenolic acids and flavonoids. The predominant compounds in the CSSE were benzoic acid (35.98%), cannabisin B (18.68%), genistein (9.55%), and epicatechin (12.92%). These findings highlight the significant presence of bioactive compounds within *C. sativa* L. seeds that may contribute to their therapeutic potential, particularly in the context of diabetes management. The predominance of benzoic acid suggests a strong antioxidant capacity, which is essential for mitigating oxidative stress associated with diabetes [28]. Additionally, the presence of flavonoids such as genistein and epicatechin is noteworthy, as these compounds have been linked to enhanced insulin sensitivity and improved glucose metabolism [29,30].

**Table 3:** Compounds identified by UHPLC-DAD–ESI/MS in CSSE

No	RT	UV	*m*/*z* (M–H)-	Proposed compounds	Subclass	% Area	Chemical structures
1	3	272	121.12	Benzoic acid	Benzoic acid	35.98	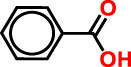
2	17.98	285.335	595.4	Cannabisin B	Lignanamide	18.68	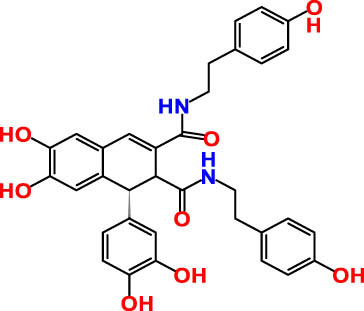
3	18.89	285.335	595.4	Cannabisin B isomer 1	Lignanamide	4.24	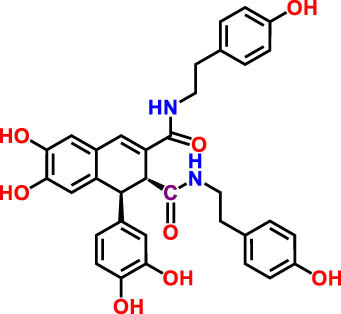
4	20.1	255	593.12	Cannabisin A	Lignanamide	4.46	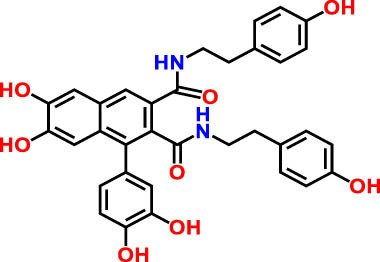
5	25.84	220.289	623.14	Cannabisin F	Lignanamide	3.85	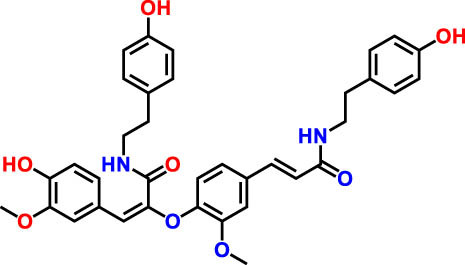
6	26.9	262	269.1	Genistein	Isoflavone	9.55	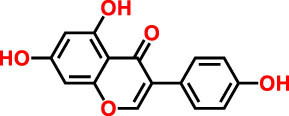
7	28.25	277	289.12	Epicatechin	Flavan	12.92	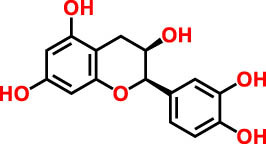

### 3.4 Effect of hydro-ethanolic extract on alloxan-induced diabetic rats

#### 3.4.1 Effect of CSSE on body weight

It is widely recognized that a decrease in body weight is one of the most straightforward indicators of diabetes [31]. Table 4 illustrates the changes in body weight for each group over a span of four weeks. Based on these findings, all diabetic groups exhibited significant weight loss compared to the normal control group during the test period, particularly after 28 days (*p* < 0.0001). In contrast, the control group demonstrated an increase in body weight. The observed decrease in body weight in diabetic rats can be attributed to the increased breakdown of fats and structural proteins, which serve as alternative energy sources due to limited carbohydrate availability.

**Table 4:** Effects of CSSE on animals’ body weights during 28 days

	Body weight (g)				
	Day 1	Day 7	Day 14	Day 21	Day 28
Normal control	214.6 ± 1.9	213.6 ± 2.15^###^	215.4 ± 1.85^####^	219 ± 2.6^####^	226.8 ± 1.6^####^
Diabetic control	219.6 ± 2.24	205.6 ± 2.44***	177.2 ± 2.31****	139.8 ± 2.63****	129 ± 3.03****
Diabetic + glibenclamide (2 mg/kg)	220.4 ± 3	212.4 ± 1.85^Ns^, ^###^	207.8 ± 1.72****, ^####^	204.6 ± 1.35****, ^####^	207 ± 2.28****, ^####^
Diabetic + CSSE 300 mg/kg	222.4 ± 2.57	212.8 ± 2.56^Ns^, ^###^	201 ± 2.89****, ^####^	195.2 ± 172****, ^####^	182.4 ± 2.33****, ^####^

Values are represented as means ± SD (*n* = 5 rats). ****p < 0.0001, ***p < 0.001, compared to normal controls; ^####^ *p* < 0.0001, ^###^ *p* < 0.001, compared to diabetic control; ns = not significant.

#### 3.4.2 Effect of CCSE on fasting blood glucose

**Figure 3:**
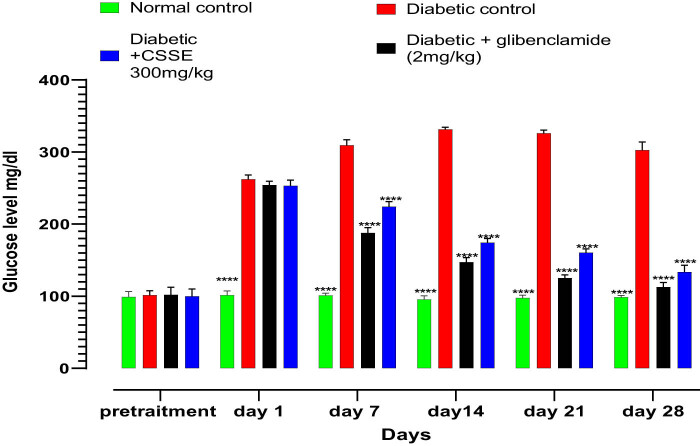
Effects of CSSE on fasting blood glucose levels in normal and alloxan-induced diabetic rats, values are represented as means ± SD (*n* = 5 rat); ****p < 0.0001, compared to DC.

Diabetes is marked by hyperglycemia, which involves elevated levels of glucose in the bloodstream. Alloxan is a well-known diabetogenic compound that specifically targets and damages pancreatic β cells, leading to their destruction and thus aiding in the development of type 1 diabetes. The fasting blood glucose levels of rats were measured before diabetes induction at days 1, 7, 14, 21, and 28, during the treatment period, as illustrated in Figure 3. All treatment groups exhibited normal blood glucose levels prior to the induction of diabetes, and there were no statistically significant differences observed among them, after diabetes induction (day 0), the glucose concentration was high significantly elevated in diabetic groups compared to the normal group.

**Figure 4:**
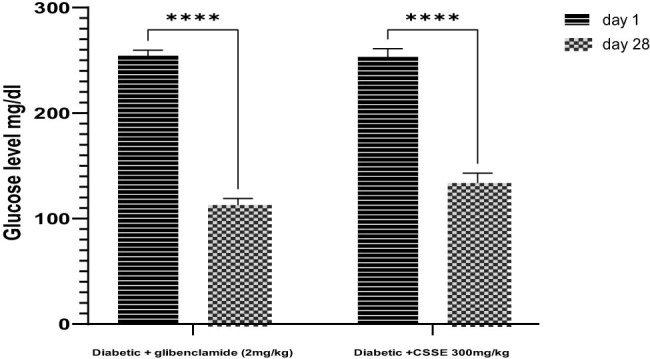
Percentage decrease in mean fasting glucose levels at day 1 and day 28 respectively, Data are presented as mean ± SD.

As evidenced by the data presented in the same figure, the daily administration of CSSE (300 mg/kg) led to a significant (*p* < 0.0001) decrease in blood glucose levels compared to the DC group starting from day 7, the same result was observed in the glibenclamide (2 mg/kg) group. Upon comparison of mean blood glucose levels on day 28 to those on day 1, a significant reduction was noted (*p* < 0.0001). The glibenclamide group displayed a 55.67% decrease, whereas the 300 mg/kg CSSE group showed a 47.30% decrease (Figure 4).

#### 3.4.3 Effect of CCSE on serum biochemical parameters

Aspartate aminotransferase (ASAT) and alanine aminotransferase (ALAT) are liver enzymes, serve as indicators of hepatocellular health, and are commonly employed to assess liver function; as shown in Table 5, the ASAT and ALAT revealed significantly heightened levels in the DC group compared to normal group (*p* < 0.0001), Conversely, in groups treated with CSSE (300 g/kg), or gilbenclamide (2 mg/kg), the rats were shielded from the rise in ASAT and ALAT levels.

**Table 5:** Effect of CCSE on kidneys and liver biochemical parameters

Groups	Liver biomarkers		Kidney biomarkers	
	ASAT (UI/L)	ALAT (UI/L)	Urea (g/L)	Creatinine (mg/L)
Normal control	113 ± 15****	67.33 ± 4****	0.58 ± 0. 2*	2.16 ± 0.18****
Diabetic control	169 ± 22	94 ± 5	0.94 ± 0.09	1.51 ± 0.29
Diabetic + glibenclamide (2 mg/kg)	141 ± 19***	80.66 ± 2.5**	0.61 ± 0.11*	1.84 ± 0.21*
Diabetic + CSSE 300 mg/kg	145 ± 12***	82 ± 3**	0.61 ± 0.07*	1.80 ± 0.13*

Values are mean ± SD (*n* = 5), *p < 0.05, **p < 0.01, ***p < 0.001, ****p < 0.0001 compared to DC.

Changes in kidney markers are demonstrated in Table 5. Elevated levels of urea and creatinine in the blood were also noted in the untreated diabetic groups; however, oral administration of CSSE (300 g/kg), or gilbenclamide (2 mg/kg) in diabetic rats, resulted in the normalization of these levels.

#### 3.4.4 Effect of CSSE on lipid profiles

**Figure 5:**
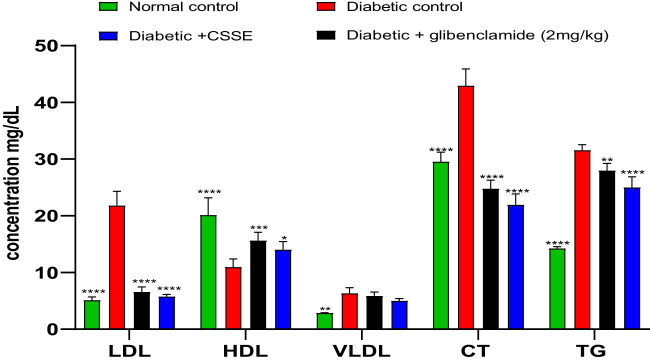
The serum concentrations of TG, CT, HDL, LDL, and VLDL. Values are presented as Means ± SD (*n* = 5), *p < 0.05, **p < 0.01, ***p < 0.001, ****p < 0.0001 compared to DC.

In this current research, we examined the lipid profile due to its connection with changes in lipid metabolism often observed in individuals with diabetes. The graphical representation in Figure 5 illustrates the serum levels of TG, total CT, HDL, LDL, and VLDL across all groups. In the diabetic rat groups, there was a notable increase in serum levels of TG, total CT, LDL, and VLDL compared to the normal control rats. Furthermore, administering *C. sativa* L. seed extract (CSSE) at a dosage of 300 mg/kg or glibenclamide at 2 mg/kg to diabetic rats for 28 days significantly reversed these increases in lipid metabolism indicators.

#### 3.4.5 OGTT

**Figure 6:**
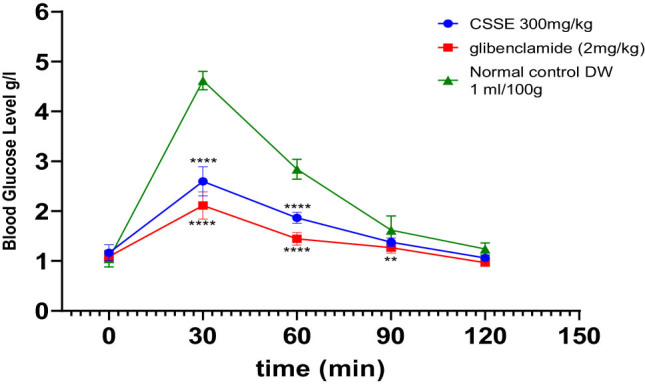
The influence of CSSE and glibenclamide on postprandial blood glucose levels in healthy rats following glucose administration 5 g/kg, values represented as mean ± SD (*n* = 5), **p < 0.01, ****p < 0.0001, compared to normal control.

The impact of hydro-ethanolic extract from *C. sativa* L. seeds (CSSE) on the OGTT in rats is depicted in Figure 6. In the untreated normal control group (1 mL/100 g body weight), there was a pronounced spike in blood glucose levels, peaking at the 30-min mark following glucose administration. In contrast, the oral intake of CSSE at a dosage of 300 mg/kg, as well as glibenclamide at 2 mg/kg, resulted in a significant reduction in blood glucose levels at the 30-min mark (*p* < 0.0001), effectively preventing hyperglycemia. Moreover, blood glucose levels remained stable at 60, 90, and 120 min post-administration compared to the control groups.

#### 3.4.6 Inhibition of α-amylase activity *in vitro*

In light of the adverse reactions and potential toxicity associated with existing medications for managing hyperglycemia, there has been a concerted effort in research to identify novel pancreatic α-amylase inhibitors derived from natural sources. This includes a focus on plants exhibiting hypoglycemic effects with minimal or no side effects. Our study aimed to evaluate the inhibitory effects of CSSE (plant extract) on pancreatic α-amylase, using acarbose as a reference drug.

The results indicated that CSSE extracts effectively inhibited α-amylase activity, with an IC_50_ value of 25.02 ± 4.03 μg/mL. Although this demonstrates significant inhibitory activity, it is noteworthy that CSSE exhibited reduced efficacy compared to acarbose, which showed an IC_50_ value of 12.33 ± 3.7 μg/mL (Table 6).

**Table 6:** α-Amylase inhibitory activities of CSSE and acarbose

	IC_50_ (μg/mL)
Acarbose	12.33 ± 3.70^a^
CSSE	25.02 ± 4.03^b^

Values in identical columns annotated with the same letter did not exhibit significant differences according to Tukey’s multiple range test (*p* < 0.05).

#### 3.4.7 Inhibition of α-amylase activity *in vivo*

In the oral starch tolerance test designed to evaluate *in vivo* α-amylase inhibition, blood glucose concentrations in all rat groups peaked (PBG) at 0.5 h post-gavage, subsequently returning to baseline levels over time. Notably, the groups receiving CSSE at a dosage of 300 mg/kg and acarbose at 10 mg/kg demonstrated significant reductions in postprandial hyperglycemia compared to the normal control group at 30 min, 60 min (****p < 0.0001), and 90 min (***p < 0.001).

**Figure 7:**
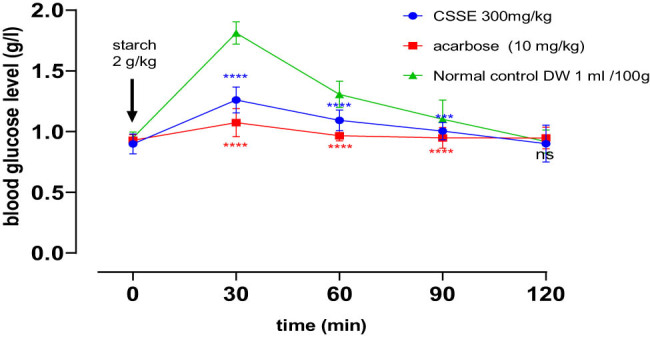
The influence of CSSE and acarbose on postprandial blood glucose levels in healthy rats following starch administration 2 g/kg, values represented as mean ± SD (*n* = 5), *p < 0.05, ***p < 0.001, ****p < 0.0001, compared to normal control.

Furthermore, it was evident that acarbose at a dosage of 10 mg/kg exhibited a more potent hypoglycemic effect than the CSSE group (300 mg/kg), as illustrated in Figure 7.

The groups administered with CSSE or acarbose exhibited a statistically significant decrease in peak blood glucose (PBG) levels and area under the curve (AUC) in comparison to the normal control group (Table 7).

**Table 7:** The impact of acarbose 10 mg/kg, and CSSE 300 mg/kg PBG and AUC following starch (2 g/kg) administration in normal rats

Groups	Peak blood glucose (PBG) (g/L)	% Diminution of PBG	AUC (g/L)	% Diminution of AUC (g/L)
Normal control	1.81 ± 0.13^a^	—	34.77 ± 4.65^a^	—
Acarbose 10 mg/kg	1.07 ± 0.22^b^	41	7.230 ± 2.32^b^	80
CSSE 300 mg/kg	1.25 ± 0.23^c^	31	13.95 ± 1.5^c^	60

Values represent the mean ± SD (*n* = 5), Values in identical columns annotated with the same letter did not exhibit significant differences according to Tukey’s multiple range test (*p* < 0.05).

### 3.5 Glide molecular docking results

Seven polphenol ligands of hydro-ethanolic acid extract of *C. sativa* seeds as well as the reference drug and the co-crystallized ligand of the chosen target protein were docked to explore their α-amylase inhibitory activity and their docking data is shown in Table 8 and Table S1. The hit compound cannabisin B shows a Glide *G*-score of −7.791 kcal/mol which is very close to the *G*-score of the standard drug for α-amylase inhibition (acarbose), i.e., −7.203 kcal/mol, and even higher than the co-crystallized ligand (−6.923 kcal/mol).

**Table 8:** Glide score, H-bonding interactions with distances (Å), polar, hydrophobic, and other interacting residues for investigated ligands with α-amylase (PDB ID: 4GQR) target protein

Title	*G*-score (kcal/mol)	Emodel (kcal/mol)	HBI residue (distance Å)	Polar residues	Hydrophobic and other interacting residues
Cannabisin B (F)	−7.791A	−92.093	GLN63 (2.18)	GLN63	TRP58, TRP59, TYR62, ALA106, LEU162, LEU165, ALA198
			ASN105 (2.29)	ASN105	
			ALA106 (2.08)	THR163	
			ASP197 (1.82)		
Acarbose (B)	−7.203B	−95.973	GLN63 (2.25)	GLN63	TRP58, TRP59, TYR62, LEU162, ALA106, LEU165, ALA198, ILE235
			ASN105 (2.67)	ASN105	
			THR163 (1.81)	THR163	
			ASP197 (1.82)	ASN298	
			GLU233 (1.92, 2.08)		
Co-crystallized ligand (4GQR) (C)	−6.923C	−63.397	GLN63 (1.82)	GLN63	TRP58, TRP59, TYR62, LEU162, LEU165, ALA198
			ASP197 (1.62, 1.80)		

**Figure 8:**
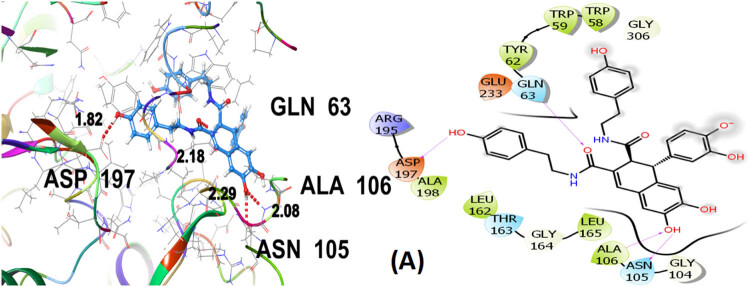
3D and 2D representation of cannabisin B (A) with α-amylase (PDB ID: 4GQR).

**Figure 9:**
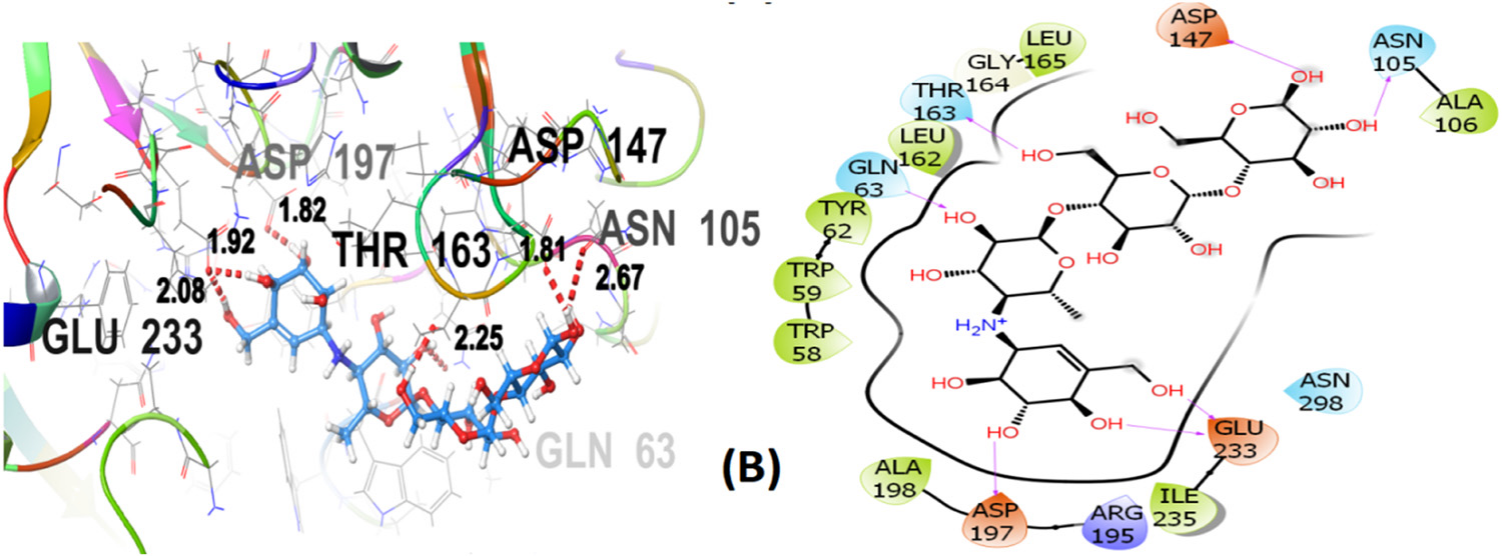
3D and 2D representation of acarbose (B) with α-amylase (PDB ID: 4GQR).

**Figure 10:**
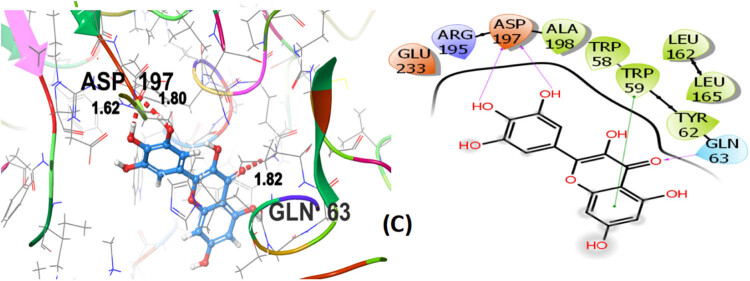
3D and 2D representation of co-crystallized ligand (C) with α-amylase (PDB ID: 4GQR).

Cannabisin B (A) as shown in Figure 8 interacts via GLN63 (2.18), ASN105 (2.29 Å), ALA106 (2.08 Å), and ASP197 (1.82 Å) amino acid residues with the target protein through hydrogen bonding. Polar interactions are contributed by GLN63, ASN105, and THR163. Diverse hydrophobic interactions are observed between the OH group of the hit compound and TRP58, TRP59, TYR62, ALA106, LEU162, LEU165, and ALA198 amino acids of the target protein. Hydrophobic contacts facilitate in binding affinity of the ligands. Acarbose, being the standard drug used in this study to evaluate the α-amylase inhibition profile of the investigated ligands, shows that GLN63 (2.25 Å), ASN105 (2.67 Å), THR163 (1.81 Å), ASP197 (1.82 Å), and GLU233 (1.92 Å, 2.08 Å) develop hydrogen bonding as depicted in Figure 9. Polar amino acids are GLN63, ASN105, THR163, and ASN298. The co-crystallized ligand of the chosen target protein shows hydrogen bonding with GLN63 (1.82 Å), and ASP197 (1.62 Å, 1.80 Å), along with the polar contacts of GLN63 as presented in Figure 10. In addition to the interactions detailed above, Figures S1–S6 illustrate the various interactions between pancreatic α-amylase (PDB ID: 4GQR) and the other ligands examined in this study.

Our findings indicate that *C. sativa* L. seed extract (CSSE) exhibits significant α-amylase inhibitory activity, reduces postprandial glucose levels, and improves lipid profiles in diabetic rats. Molecular docking studies further supported these findings, revealing strong interactions between identified phenolic and the α-amylase enzyme.

## 4 Discussion

The significant public health challenge posed by diabetes mellitus has prompted an ongoing search for effective preventive alternatives. *C. sativa* L., recognized as a medicinal plant, has been utilized in traditional African medicine for centuries, particularly in managing chronic diseases such as diabetes [32,33] This investigation assessed the impact of a hydro-ethanolic extract derived from *C. sativa* L. seeds on diabetes and its associated complications.

As detailed in Section 3, the plant extract exhibited favorable effects on all physiological parameters evaluated in rats induced with diabetes via alloxan.

The quality assessment of the cannabis seed extract (CSSE) was conducted by evaluating various parameters. The oil content observed in this study aligns closely with previous research findings. For instance, Mihoc et al. [34] reported similar oil content in five Romanian varieties, while Taaifi et al. investigated Moroccan *C. sativa* L. seeds from diverse locations and found oil content ranging from 26.42 to 35.19% [35]. Additionally, Munteanu et al. documented a lipid content of 29.17 ± 0.46%, which is consistent with our findings [9].

The ash content recorded in this study exceeded previously reported values by Taaifi et al. [35] but was consistent with findings from Munteanu et al. and Bhatt et al. [9,36] while being lower than those obtained by Rodriguez-Martin et al. [37]. Furthermore, screening results for phytochemicals indicate that *C. sativa* L. seeds are rich in secondary metabolites, underscoring their significance as a medicinal plant. Numerous studies have highlighted that phytochemicals play a crucial role in exhibiting antioxidant, anti-hyperglycemic, and anti-hyperlipidemic effects. For example, tannins, phenols, and saponins inhibit glucose transport by blocking the sodium-glucose co-transporter-1 (S-GLUT-1) and hinder carbohydrate digestion by inhibiting α-amylase and α-glucosidase [38,39].

Our analysis identified seven polyphenols as major constituents of CSSE, aligning with multiple studies that have identified specific phenolic compounds in *C. sativa* seeds [9,40,41,42,43]. Notably, Martinez and collaborators reported the presence of genistein and epicatechin in extracts of *C. sativa* seeds [42]. Benkirane et al. also identified several lignanamides in defatted hempseeds, including cannabisin C, D, and M, along with phenolic acids such as *p*-coumaric acid and ferulic acid [44] Furthermore, Haddou et al. noted that 3-hydroxycinnamic acid was the major compound in aqueous extracts of hemp seeds [45]. In addition to these findings, Ahmad et al.’s phytochemical investigation successfully identified benzoic acid, gallic acid, and kaempferol as primary phenolic components in methanolic extracts of *C. sativa* seeds [46]. The abundance of phenolic compounds in CSSE could potentially contribute to diverse pharmacological activities.

Diabetes induced by alloxan leads to the degeneration and shrinkage of pancreatic beta cells through excessive formation of reactive oxygen species, resulting in insulin deficiency [47]. This condition creates a complex pathophysiological state characterized by significant weight loss and increased intake of water and food alongside elevated urine output [48]. Studies indicate that increased water consumption and diuresis are directly linked to hyperglycemia due to absolute or relative insulin deficiency leading to elevated blood glucose levels [49,50]. Consequently, the kidneys expel excess sugar through urine water follows glucose due to its osmotic effect stimulating thirst to offset water loss [51].

The protective effect of CSSE against body weight loss may be attributed to its ability to increase insulin levels, thereby improving glycemic control. A 28-day treatment with CSSE exhibited significant antihyperglycemic effects consistent with those observed by Munteanu et al., who studied the *C. sativa* L. zenith variety [9]. It has been demonstrated that fixed oils from *C. sativa* L. exert hypoglycemic effects on diabetic rats [52]. Additionally, the antihyperglycemic impact of cannabis essential oil has also been documented, reinforcing the potential of various cannabis-derived products in diabetes management [53].

The liver plays a crucial role in glucose homeostasis and xenobiotic metabolism; thus, concentrations of ALT and AST in the bloodstream serve as reliable indicators of liver function status. Previous studies have shown necrosis in the liver of alloxan-induced diabetic rats [54]. Elevated activities of ALT and AST are primarily linked to their release from liver cytosol into circulation due to cellular damage [49]. Notably, administering CSSE alongside glibenclamide to alloxan-diabetic groups for 28 consecutive days restored enzyme activities to baseline levels.

Diabetic animals typically exhibit significantly higher serum levels of urea and creatinine – critical markers for renal impairment [55]. The substantial decrease in serum urea and creatinine levels among diabetic rats treated with CSSE and glibenclamide suggests its potential role in halting renal damage associated with diabetes progression.

Moreover, diabetes mellitus is widely recognized as a prevalent factor contributing to cardiovascular diseases due to its association with significant changes in plasma lipids [56] Insulin regulates lipid metabolism; impaired insulin signaling can lead to hypertriglyceridemia and hypercholesterolemia characterized by elevated VLDL and LDL levels while decreasing HDL concentrations [48]. In diabetic states, hypercholesterolemia arises because insulin inhibits β-hydroxy-β-methylglutaryl-coenzyme-A reductase an essential enzyme involved in cholesterol metabolism [39]. Furthermore, diabetes renders lipoprotein lipase inactive, an enzyme crucial for triglyceride breakdown. This inactivity results in elevated triglyceride levels and a decline in HDL levels [57]. CSSE demonstrated hypocholesterolemic and hypotriglyceridemic effects similar to antihyperlipidemic medications that lower lipid levels while increasing HDL cholesterol concentrations [9,58]. An effective anti-diabetic agent should control glucose level rises through various mechanisms; thus, we assessed this using oral glucose-loaded hyperglycemic models where CSSE proved effective. An effective antidiabetic agent should control glucose level rises through various mechanisms; thus, we assessed this using oral glucose-loaded hyperglycemic models where CSSE proved effective.

Lowering postprandial hyperglycemia is crucial for diabetes management achieved by inhibiting α-amylase an enzyme that breaks down complex polysaccharides into smaller oligosaccharides through hydrolysis [59]. α-Amylase inhibition leads to a reduction in the formation of oligosaccharides and disaccharides, ultimately resulting in decreased glucose absorption into the bloodstream. Consequently, postprandial blood glucose levels are effectively lowered. Our findings indicate that CSSE exhibits significant α-amylase inhibition with an IC_50_ value of 25.02 ± 4.03 µg/mL. Molecular docking studies further supported these findings, revealing strong interactions between identified phenolic and the α-amylase enzyme. While previous studies on the effects of *C. sativa* on α-amylase yielded mixed results Zengin et al. reported no significant effects while Haddou et al. demonstrated promising α-amylase inhibitory activity [60,61]. However, Leonard et al. reported that a *C. sativa* L. seed hull extract did not exhibit significant α-amylase inhibitory activity [40]. These discrepancies may stem from variations in plant variety, growth conditions, extraction methods, or specific compounds present within the extracts. The observed α-amylase inhibition indicates that further exploration of CSSE’s role in diabetes management is warranted.

α-Glucosidase also plays a vital role in carbohydrate digestion by acting on glycosidic bonds to produce glucose [62]. Inhibiting α-glucosidase is advantageous for managing pre-diabetic and diabetic conditions as it plays a critical role in carbohydrate absorption within the gastrointestinal tract [40]. Earlier research indicated that ethanolic extracts from *C. sativa* seeds demonstrate α-glucosidase inhibitory activity [63,64] aligning with Ren’s findings regarding oligopeptides derived from *C. sativa* L. seeds protein exhibiting potent α-glucosidase inhibition activity [65]. Supporting these findings, Leonard et al. [40] concluded that phenolic extracts from *C. sativa* L. seeds showed α-glucosidase inhibitory activity comparable to the pharmaceutical standard, acarbose. This collective evidence highlights the potential of *C. sativa* seed extracts and derivatives in the management of carbohydrate metabolism disorders.

*C. sativa* seed extract exhibits high antioxidant potential [43,44,66,67,68] due to its richness in phenolic compounds and fatty acids [69,70]. This antioxidant property aids in preventing the degeneration of pancreatic islets, preserving their histoarchitecture. Additionally, because a lower dose of alloxan induces only partial destruction of pancreatic beta cells, the surviving beta cells in albino rats can regenerate [71].

Beyond their antioxidant properties, phenolic compounds from various plant sources have been shown to possess therapeutic benefits for diabetes management [13]. These compounds can enhance insulin secretion, reduce glucose production by the liver, inhibit key enzymes involved in carbohydrate digestion, improve the sensitivity of peripheral tissues to insulin, and regulate glucose absorption in the bloodstream. Collectively, these actions contribute to improved post-meal blood sugar control [48,72].

Phenolic compounds in *C. sativa* L. seed extract stimulate postprandial insulin secretion, promote L cells and K cells to secrete glucagon-like peptide-1 (GLP-1) and glucose-dependent insulinotropic polypeptide (GIP) [73], and increase their half-life by inhibiting dipeptidyl peptidase-4 (DPP-4) [74,75]. Additionally, they stimulate the expression of glucose transporters (GLUT) types 2 and 4 in the liver, skeletal muscle, and adipose tissues [76], enhancing the binding of insulin to insulin receptors [30,77,78]. Benzoic acid, genistein, and epicatechin, the major compounds in CSSE, reduce β-cell apoptosis, regenerate damaged pancreatic islets, and enhance insulin secretion from surviving beta cells, resulting in an antihyperglycemic effect [79,80,81,82]. Lignanamides, such as cannabisins, found in *C. sativa* L. seeds exhibit antioxidant and anti-inflammatory activities, which may contribute to their beneficial effects on glucose metabolism [83,84,85]. However, specific studies directly linking lignanamides and cannabisins to diabetes management are limited. Further research is needed to fully elucidate their mechanisms of action and therapeutic potential in the context of diabetes. Cannflavins, unique prenylated flavonoids found in cannabis seeds, exhibit antioxidant, anti-inflammatory, and anticancer properties, which may also contribute to their potential benefits in diabetes management [86,87,88].

The anti-diabetic properties of CSSE may result from a synergistic effect of multiple bioactive molecules instead of a single compound.

## 5 Conclusions

Our findings demonstrate that *C. sativa* L. seed extract (CSSE) holds significant promise as a novel therapeutic approach for managing diabetes and its associated complications. The extract’s rich phenolic profile, particularly compounds like benzoic acid, cannabisin B, genistein, and epicatechin, contributes to its potent α-amylase inhibitory activity. This, coupled with its hypoglycemic, hypolipidemic, hepatoprotective, and nephroprotective effects observed in our *in vivo* studies, suggests its potential to improve glycemic control and overall metabolic health. Furthermore, *in silico* docking studies validate the strong binding affinity of these phenolic compounds to the α-amylase enzyme, providing mechanistic insights into their inhibitory activity.

The clinical implications of our findings are substantial. By inhibiting α-amylase, CSSE can delay carbohydrate digestion and absorption, leading to a more gradual increase in blood glucose levels. This could be particularly beneficial for individuals with type 2 diabetes, who often experience postprandial hyperglycemia. Additionally, the hypolipidemic and antioxidant properties of CSSE may contribute to the prevention of diabetic complications, such as cardiovascular disease and neuropathy.

While our findings are promising, further research, including well-designed clinical trials, is necessary to fully evaluate the safety and efficacy of CSSE in human subjects. By elucidating the underlying molecular mechanisms, we can optimize the therapeutic potential of this natural product and develop innovative strategies for diabetes management.

## Supplementary Material

Supplementary material
