# Supplementary material for: Characterization of phenolic compounds and evaluation of anti-diabetic potential in Cannabis sativa L. seeds: In vivo, in vitro, and in silico studies
Source: Open Life Sci. 2024 Dec 31;19(1):20221024. doi: 10.1515/biol-2022-1024 (PMC11736388; doi:10.1515/biol-2022-1024)
Supplement: Supplementary material [file biol-2022-1024-sm.pdf]

Supplementary material

Table S1

Glide molecular docking results

The ligand cannabisin A (Figure S1) engages in hydrogen bonding and hydrophobic interactions with the target protein. It form hydrogen bonding with relative distances in angstroms as GLN63 (2.23 Å), ASP197 (1.74 Å), ASP356 (2.75 Å) which are responsible to contribute to the ligand's specific positioning near the binding site. Polar interactions are contributed by GLN63, and THR163 amino acid residues. Hydrophobic interactions involve PRO54,TRP58, TRP59,TYR62, LEU162,LEU165, ALA198,TRP357. These interactions are critical to stabilize the ligand within the binding pocket. The GScore is −6.975 kcal/mol. Cannabisin B isomer 1 (Figure S2) interacts hydrophobically with TRP58,TRP59, TYR62, ALA106, VAL107,TYR151,LEU162, LEU165,ILE235,PHE256, ALA307,TRP357 resulting in GScore of −6.85 kcal/mol. Polar amino acid residue involved are GLN63 THR163, HIS201, ASN298 whereas GLU233 (1.59 Å), ASP195 (2.78 Å), ASP197 (1.58Å), ASP356 (1.69 Å) have established the H-bond. Epicatechin (Figure S3) interacts with TRP58,TRP59, TYR62,LEU162, LEU165,ALA19, ILE235 via hydrophobic contacts as well as the polar interactions are evident with GLN63, THR163, and HIS201 amino acid residues along with the formation of hydrogen bonding by TRP59 (2.72), THR163 (2.67 Å), ASP197 (2.29 Å), GLU233 (2.60 Å). Its relevant GScore is −6.831 kcal/mol. Similarly, Genistein (Figure S4) interacts hydrophobically with TRP58,TRP59, TYR62, LEU165,ALA19, ILE235 resulting in docking score of −5.949 kcal/mol. GLN63, THR163, and ASN298 are involved in polar interactions whereas GLU233 (1.65 Å), ASP197 (2.05 Å) respectively show

Table S1: Glide Score, H-Bonding Interactions With Distances (Å), Polar, Hydrophobic, and Other Interacting Residues for Investigated Ligands With α-amylase (PDB ID: 4GQR) Target Protein

Title	G-score (kcal/ mol)	Emodel (kcal/ mol)	HBI residue (distance Å)	Polar residues	Hydrophobic and other interacting residues
Cannabisin A (a)	−6.975	−84.805	GLN63 (2.23)<br>ASP197 (1.74)<br>ASP356 (2.75)	GLN63<br>THR163	PRO54, TRP58, TRP59, TYR62, LEU162, LEU165, ALA198, TRP357
Cannabisin B isomer 1 (b)	−6.85	−92.853	GLU233 (1.59)<br>ASP195 (2.78)<br>ASP197 (1.58)<br>ASP356 (1.69)	GLN63<br>THR163<br>HIS201<br>ASN298	TRP58, TRP59, TYR62, ALA106, VAL107, TYR151, LEU162, LEU165, ILE235, PHE256, ALA307, TRP357
Epicatechin (c)	−6.831	−60.44	TRP59 (2.72)<br>THR163 (2.67)<br>ASP197 (2.29)<br>GLU233 (2.60)	GLN63<br>THR163<br>HIS201	TRP58, TRP59, TYR62, LEU162, LEU165, ALA19, ILE235
Genistein (d)	−5.949	−52.713	GLU233 (1.65)<br><br>ASP197 (2.05)	GLN63<br>THR163<br>ASN298	TRP58, TRP59, TYR62, LEU165, ALA198, ILE235
Cannabisin F (e)	−5.393	−72.195	ASP147 (1.81)	GLN63<br>THR163	TRP58, TRP59, TYR62, ILE148, TYR151, LEU162, LEU165, ALA198, ILE235
Benzoic acid (f)	−4.574	−18.809	GLN63 (1.83)	GLN63<br>THR163	TRP59, TYR62, LEU162, LEU165

hydrogen bonding. In case of the ligands Cannabisin F and benzoic acid, (Figures S5 and S6) interacts via polar interactions with GLN63, and THR163 resulting in the docking score of −5.393 and −4.574 kcal/mol respectively. ASP147 (1.81 Å,) GLN63 (1.83 Å)are the H-bond forminf residues in s Cannabisin F and benzoic acid respectively. Cannabisin F depicted hydrophobic interactions with where as the said hydrophobic contacts are shown by TRP59, TYR62, LEU162,LEU165 in case of benzoic acid.

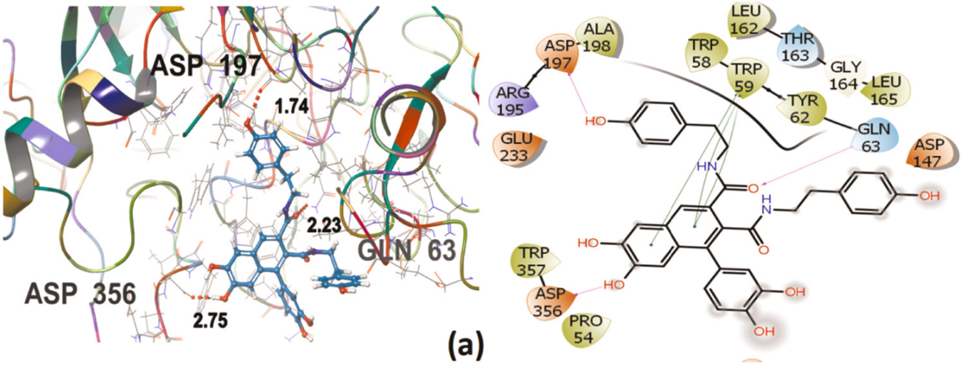

Figure S1: 3D and 2D representation of Cannabisin A (a) with α-amylase (PDB ID: 4GQR).

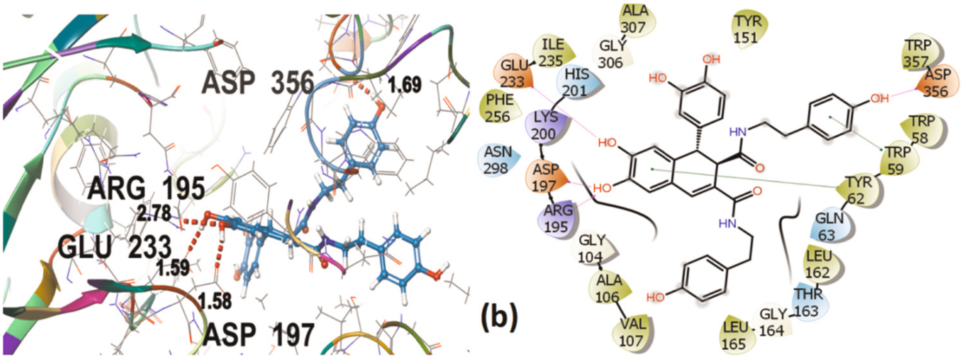

Figure S2: 3D and 2D representation of Cannabisin B isomer 1 (b) with α-amylase (PDB ID: 4GQR).

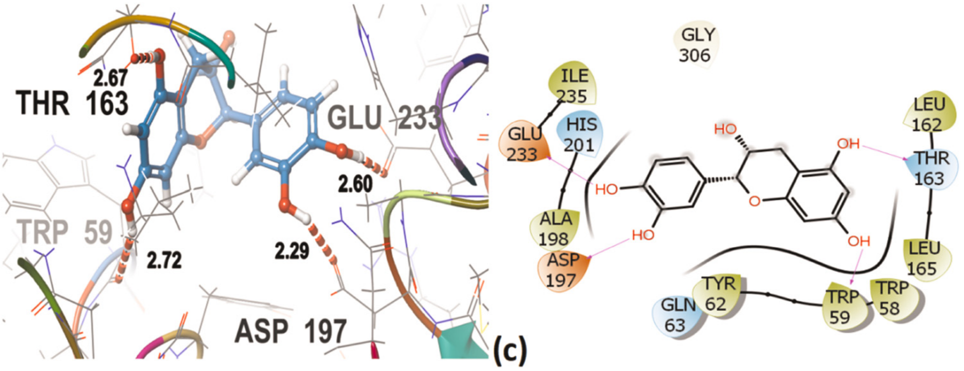

Figure S3: 3D and 2D representation of Epicatechin (c) with α-amylase (PDB ID: 4GQR).

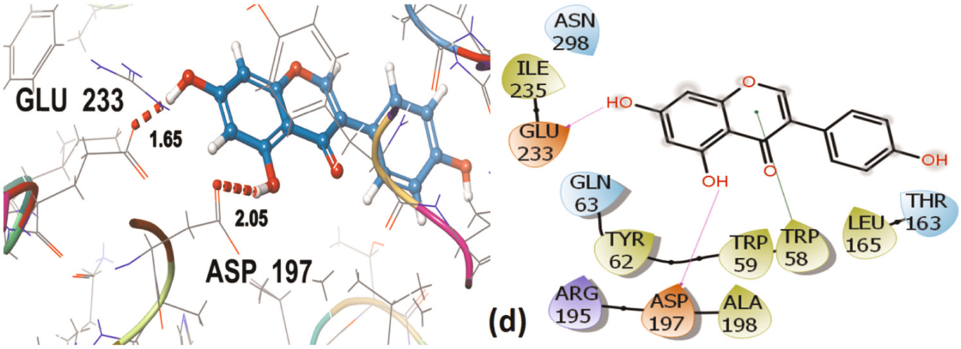

Figure S4: 3D and 2D representation of Genistein (d) with α-amylase (PDB ID: 4GQR).

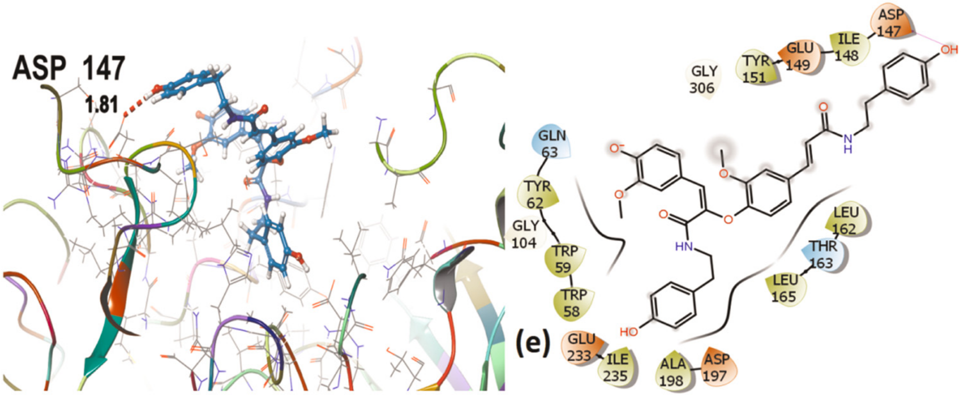

Figure S5: 3D and 2D representation of Cannabisin F (e) with α-amylase (PDB ID: 4GQR).

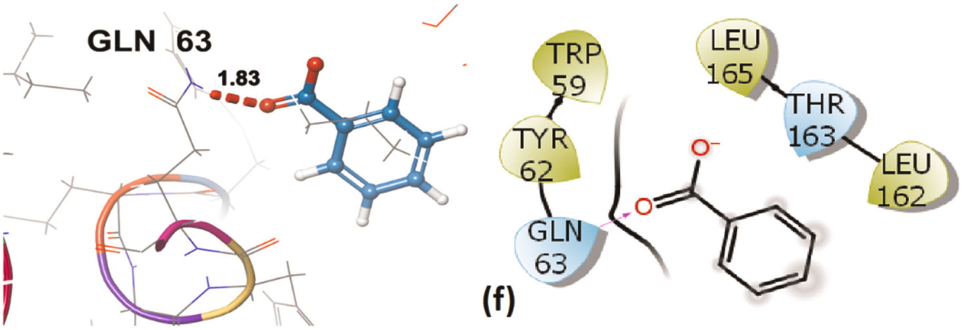

Figure S6: 3D and 2D representation of Benzoic acid (f) with α-amylase (PDB ID: 4GQR).
